# Supplementary material for: Mobilising Cross-Sectoral Collaboration in Creating Age-Friendly Cities: Case Studies from Akita and Manchester
Source: Int J Environ Res Public Health. 2025 Jan 8;22(1):73. doi: 10.3390/ijerph22010073 (PMC11764577; doi:10.3390/ijerph22010073)
Supplement: Supplementary file 1 [file ijerph-22-00073-s001.zip › ijerph-3315845-supplementary.pdf]

# Ageing in Place in Cities - Interview Schedule

**Introduction**
As a bit of background, the Ageing in Place in Cities project is a five-year research programme exploring the relationship between population ageing and urban change. The research is examining the role of 'age-friendly' policies and initiatives in shaping the experience of ageing in place.

We have already collected City Templates for each of the cities. The data in the city templates includes: statistics about the demographics of each city; narratives about the history of the city, including the development and delievery of age-related policys and programmes; and also some examples of work carried out in [your city]. So we can talk about some of this information as we go through the interview.

We expect this interview to last about 45 minutes. We will cover: a general discussion about your role and how you see [your city], what is unique or important about [your city]; we will then talk in more detail about ageing work and the policies and initiatives in the city; we will also discuss the different people and stakeholders working on ageing work; then we would like to explore a bit more about the older people in [you city] and how they are engaged in work supporting ageing in place. Finally we will like to hear your thoughts about the future, and perhaps what you think future research should focus on.

**Part A: Context**

A1. To start, can you tell us a bit about your role, what do you do? What led you to doing this work? Why do you do it, what drives you wanting to do this work?
A2. What do you think are the key characteristics of [your city]? What have been some of the demographic changes (including but not restricted to population ageing? How do these charateristics relate to the challeges and opportunities within [your city]?
A3. How are older people viewed in [your county]? How would you describe attitudes towards older people in [your city/country]?

**Part B: Policies and initiatives**

Next we are going to move on to exploring policy development within [your city]. In our research programme, we are interested in learning about how older people can be supported to age well in place, (or to grow older in their own homes and communities). We will be talking about the history of ageing work in [your city], the priorities, or the focus, of the work, and how older people are supported to age in place.

B1. What does 'ageing in place' mean to you? How does it relate to your work? Is it a term that is used (how does it translate in the local language)?
B2. Can you tell us about how the ageing agenda has developed over time in [your city]? To what extent is ageing in place a policy focus?
B3. Within the ageing agenda, what would you say has been the priority for [your city]? (health, transport, housing - could refer to Age-Friendly domains)
B4. Can you give some examples of key projects that have been carried out to support ageing in place?
B5. What would you consider to be the most important policy that supports ageing in place? (this may be national/regional/city – any policies that are relevant for older people ageing in place in the cities) Why do you think it is so important?

B6. How is impact measured or evaluated? What sytems or structures are in place to monitor success?

**Part C: Stakeholders and actors**

Next we're going to explore a few questions about who you work with and the different stakeholders in ageing work in [your city].

C1. Who do you work with on ageing work? Who else works on the ageing agenda, both in developing and delivering initiatives? (local government, community organisations, private companies, researchers, older people)
C2. How well do the various people and organisations involved in the age-friendly agenda work together?
C3. Who champions or leads the work in [your city]?
C4. How would you describe the political support for work on the ageing agenda? Where does the political support come from (national, regional, local)? What extra support do you thin is needed?

**Part D: Equity, diversity and co-production**

Now we would like to explore a bit more about the older people in [you city] and how they are engaged in work support ing ageing in place.

D1. Do you feel there are any groups within the older population that are not as involved in the age-friendly agenda, or groups you would like to engage with further?
D2. How does the work around the ageing agenda recognise and cater to differences within the older population?- ask for examples here?
D3. How are older people involved in decision-making and the development of age-friendly policy? What are the mechanisims? Does this include marginalised groups of older people? (or the groups refered to in D1)
D4. Is there support in [your city] for bringing together different groups of older people to work collectively on the ageing agenda?

**Part E: The future**

Finally, we are very interested in your thoughts about the future and what you think are some of the opportunities to support ageing in place going forward.

E1. What do you think will be the priorities going forward, which areas of work offer promising developments in terms of supporting ageing in place?
E2. Do you think there are any specific opportunities for for future of supporting ageing in place in [your city]?
E3. How would you like to see the World Health Organisation and the Global Network of Age Friendly Cities and Comm unities support your work?
E4. What would you need/expect/like to learn from being a member/ being involved in our research project?

And lastly, who else can we speak to to learn more about ageing in place policies and initiatives in your city?

Thank you for taking part, we will keep in touch if that is ok.
